# Machine learning integration of MRI and gait reveals mobility phenotypes in multiple sclerosis

**DOI:** 10.1093/braincomms/fcaf381

**Published:** 2025-10-06

**Authors:** Hernan Inojosa, Wanqi Zhao, Judith Wenk, Dirk Schriefer, Stefanie Fischer, Peter Heisig, Maren Kählig, Hannes Schlieter, Heidi Stölzer-Hutsch, Isabel Voigt, Annika Kather, Rocco Haase, Katja Akgün, Hagen H Kitzler, Katrin Trentzsch, Uwe Aßmann, Karsten Wendt, Tjalf Ziemssen

**Affiliations:** Center of Clinical Neuroscience, Department of Neurology, Medical Faculty and University Hospital Carl Gustav Carus, TUD Dresden University of Technology, Dresden 01307, Germany; Chair of Software Technology, TUD Dresden University of Technology, Dresden 01062, Germany; Center of Clinical Neuroscience, Department of Neurology, Medical Faculty and University Hospital Carl Gustav Carus, TUD Dresden University of Technology, Dresden 01307, Germany; Center of Clinical Neuroscience, Department of Neurology, Medical Faculty and University Hospital Carl Gustav Carus, TUD Dresden University of Technology, Dresden 01307, Germany; Center of Clinical Neuroscience, Department of Neurology, Medical Faculty and University Hospital Carl Gustav Carus, TUD Dresden University of Technology, Dresden 01307, Germany; Chair of Software Technology, TUD Dresden University of Technology, Dresden 01062, Germany; Chair of Software Technology, TUD Dresden University of Technology, Dresden 01062, Germany; Chair of Software Technology, TUD Dresden University of Technology, Dresden 01062, Germany; Center of Clinical Neuroscience, Department of Neurology, Medical Faculty and University Hospital Carl Gustav Carus, TUD Dresden University of Technology, Dresden 01307, Germany; Center of Clinical Neuroscience, Department of Neurology, Medical Faculty and University Hospital Carl Gustav Carus, TUD Dresden University of Technology, Dresden 01307, Germany; Center of Clinical Neuroscience, Department of Neurology, Medical Faculty and University Hospital Carl Gustav Carus, TUD Dresden University of Technology, Dresden 01307, Germany; Center of Clinical Neuroscience, Department of Neurology, Medical Faculty and University Hospital Carl Gustav Carus, TUD Dresden University of Technology, Dresden 01307, Germany; Center of Clinical Neuroscience, Department of Neurology, Medical Faculty and University Hospital Carl Gustav Carus, TUD Dresden University of Technology, Dresden 01307, Germany; Department of Neuroradiology, Medical Faculty and University Hospital Carl Gustav Carus, TUD Dresden University of Technology, Dresden 01307, Germany; Center of Clinical Neuroscience, Department of Neurology, Medical Faculty and University Hospital Carl Gustav Carus, TUD Dresden University of Technology, Dresden 01307, Germany; Chair of Software Technology, TUD Dresden University of Technology, Dresden 01062, Germany; Chair of Software Technology, TUD Dresden University of Technology, Dresden 01062, Germany; Center of Clinical Neuroscience, Department of Neurology, Medical Faculty and University Hospital Carl Gustav Carus, TUD Dresden University of Technology, Dresden 01307, Germany

**Keywords:** multiple sclerosis, multimodal analysis, quantitative MRI, mobility phenotyping, unsupervised clustering

## Abstract

Mobility impairment is a hallmark of disease worsening in multiple sclerosis (MS), yet its phenotypic diversity and pathophysiology mechanisms are not completely understood. Conventional gait assessments often rely on subjective clinical measures, which may not fully capture the complexity of gait abnormalities. The integration of advanced quantitative gait analysis, quantitative from MRI, and machine learning (ML) may reveal unique mobility phenotypes, potentially reflecting underlying disease mechanisms and heterogeneity. In this study, we aimed to identify and characterize mobility phenotypes among people with MS (pwMS) using a mixed approach with spatiotemporal gait parameters and MRI-derived features, supported by unsupervised ML clustering. 1026 pwMS underwent comprehensive gait assessments and quantitative MRI between 2018 and 2023. Principal component analysis was applied for dimensionality reduction and k-means clustering to identify distinct phenotypes. Clusters were compared using demographic, clinical, and MRI features, with statistical comparisons performed using Kruskal–Wallis and Chi-square tests. Four gait clusters were identified. Cluster 1 (faster stable, 47.8%), demonstrated the most efficient gait features and highest grey matter fractions. Cluster 4 (slow severely unstable, 7.4%) showed profound disability, shortest strides, lowest velocity, and greatest variability. Intermediate clusters 2 (slower stable, 32.3%) and 3 (moderately unstable, 12.6%) had similar velocity but differed in cadence and stride length. Cluster 3, marked by shorter steps and increased cadence, showed higher lesion burden and lower brain parenchymal fraction, suggesting emerging structural impairment and possible compensatory gait. Clinical measures aligned with these findings: unstable Clusters 3 and 4 had the highest proportion of progressive MS, worst disability scores, longest disease duration, and greatest self-reported gait impairment. Integrating quantitative MRI metrics with spatiotemporal gait analysis has the potential to phenotype clinical impairments in pwMS. ML-driven analysis identified a novel intermediate cluster, distinguished by a gait with increased cadence and shorter strides, alongside distinct MRI abnormalities. This pattern may reflect a potential adaptation within the mobility spectrum, not yet conclusively discernible by human raters but detectable through ML.

## Teaser

Inojosa *et al*. report that integrating quantitative MRI metrics with spatiotemporal gait analysis in people with multiple sclerosis identified four distinct mobility phenotypes using machine learning. This data-driven approach identifies subtle gait impairments and compensatory patterns, offering new insight into disease-related motor dysfunction and potential targets for early intervention.

## Graphical Abstract

**Graphical Abstract:**
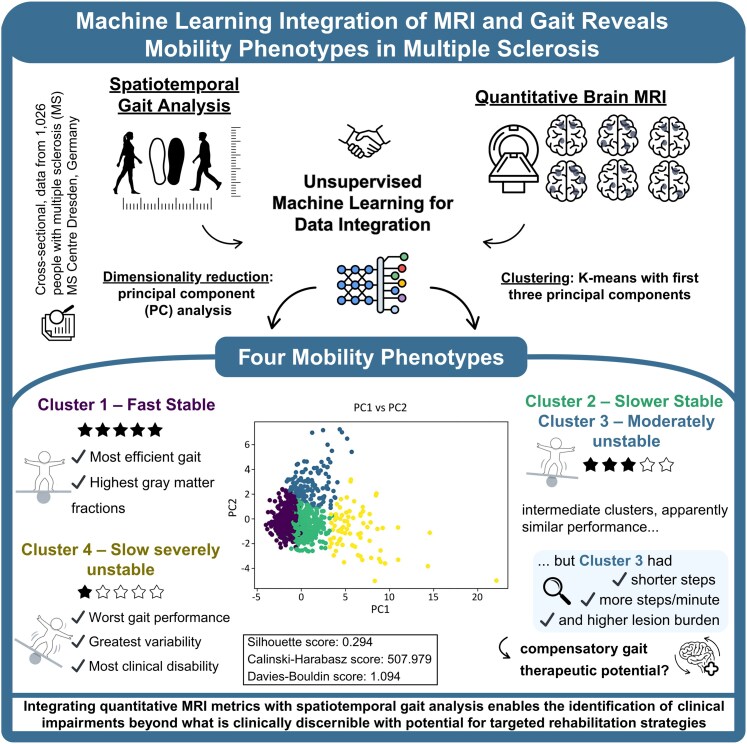

## Introduction

Gait impairment is one of the most prevalent and disabling manifestations of several neurological diseases, including multiple sclerosis (MS). This may affect over 50% to 85% of people with MS (pwMS) throughout the disease course, impacting quality of life, independence, and increasing fall risk.^1,2^ Varying patterns of central nervous system involvement may differentially affect gait in MS.^3-5^ However, integrative gait and imaging assessment is still unavailable in clinical practice.

Despite its clinical relevance, measuring gait is challenging to measure objectively.^6^ Conventional tools like the Expanded Disability Status Scale (EDSS) rely on subjective clinical evaluation and may lack responsiveness to subtle gait changes.^7,8^ Consequently, there is an increasing need for advanced technologies to capture subtle or granular deficits, including spatiotemporal parameters.^9-12^ Moreover, artificial intelligence automated MRI-based quantitative measures that offer deeper insights into MS pathology.^13,14^

Recent advances may help redefine gait assessment through deep phenotyping approaches, paving the way for targeted intervention strategies.^15^ With expanding multimodal gait and imaging datasets, machine learning (ML) offers new opportunities to uncover multidimensional MS features and previously unrecognized disease patterns.^16-18^ Unsupervised ML techniques can analyse complex multidimensional data to detect distinct profiles, potentially reflecting varying degrees of impairment, compensation strategies, or disease mechanisms.^19,20^ This may offer deeper insights into the relationship between structural changes and functional outcomes in MS, possibly uncovering hidden disease subtypes and biomarkers without the need for prior labelling which yet is often a prerequisite when taking a data-driven approach to precision medicine.^15^

Despite the potential of these technologies, the integration of ML into gait and imaging analysis to phenotype MS remains relatively underexplored. Existing studies often focus on individual gait parameters or labelled clinical outcomes.^21-24^ Dimensionality reduction techniques have been used for identifying distinct gait domains related to fallers among pwMS or patterns of gait instability.^25,26^ A promising study by Filli *et al*. identified distinct gait profiles in pwMS using innovative clustering approaches.^25^ Interestingly, clinical changes after 4 years differed across the detected gait profiles.^26^ However, in these studies, ML-driven analysis primarily focused on gait parameters without considering MRI-derived features.

In this study, we applied clustering methods to spatiotemporal gait data integrated with MRI-derived features. We aimed to identify clinically meaningful distinct gait clusters, characterize their profiles, and explore their potential implications for understanding gait heterogeneity in MS. Leveraging advanced gait and quantitative imaging analytics in MS, we propose a framework to deepen mobility phenotyping translatable to other neurological diseases and inform future research and clinical applications.

## Materials and methods

All data analysed in this study were obtained retrospectively from a local cohort participating in the Multiple Sclerosis Partners Advancing Technology and Health Solutions (MS PATHS) project.^27^ PwMS were assessed at the MS Center Dresden between 1 January 2018, and 1 May 2023. The dataset includes participants with a confirmed MS diagnosis according to the 2017 McDonald criteria who were aged 18 years or older and who were able to walk with or without a walking assistive device.^28^ Participants with a history of non-MS-related neurological or orthopaedic conditions affecting gait were excluded. MS PATHS participants were recruited during routine clinical visits, where gait analysis was performed as part of a standardized assessment protocol following the Dresden Protocol for Multidimensional Walking Assessment.^9^ All participants had provided written informed consent prior to enrolment. The study was conducted in accordance with the Declaration of Helsinki and approved by the Ethics Committee of Technische Universität Dresden (BO-EK-580222017 and BO-EK-399092023).

### Quantitative gait analysis

Gait performance was assessed using the GAITRite instrumented walkway (CIR Systems Inc., Franklin, NJ, USA), a validated system that captures spatiotemporal gait parameters with high precision and reliability.^11^ The GAITRite system consists of an electronic walkway embedded with pressure-sensitive sensors that measure key gait metrics in real-time. Participants walked at their self-selected comfortable speed across the walkway, allowing for the collection of multiple gait cycles. A mean of 37.2 [standard deviation (SD) 15.4)] valid steps were analysed per gait assessment.

The system captures essential gait features, including both direct and derived measures such as velocity (cm/s), cadence (steps/min), base of support (cm), stride length (cm), step time (s), swing time variability (s), stride length variability (cm), step time differential (s), stance time differential (s), swing time (s), step time (s), and double support time (s). Variability measures were expressed as SD.

### Brain imaging

MRI data were acquired using standardized imaging protocols on 3-T Siemens scanners. The imaging sequences included 3D T1-weighted and 3D FLAIR sequences with 1-mm isotropic voxels, ensuring high-resolution volumetric assessment and lesion detection. Volumetric and lesion-specific features were derived, including, among others brain parenchymal fraction (BPF), total and infratentorial T2 lesion volume, or thalamic volume.

The standardized imaging protocol ensured consistency across scans, facilitating reliable extraction of quantitative features relevant to brain atrophy and lesion burden.^27^ Brain MRI data closest in time to the gait analysis session were used, with a time difference ranging from 0 to a maximum of 180 days (median 69 days, IQR 36–123 days), ensuring temporal alignment between imaging and clinical assessments.

### Clinical measures

Clinical measures were documented at the time of gait analysis, including demographic characteristics, MS clinical classification [relapsing-remitting multiple sclerosis, primary progressive multiple sclerosis and secondary progressive multiple sclerosis], and key clinical outcomes [e.g. disease duration, disease modifying treatment (DMT)]. Participants were evaluated by Neurostatus-certified neurologists, and disability was assessed using the EDSS.^7^ The EDSS is a validated tool for quantifying disability in MS, ranging from 0 (normal neurological function) to 10 (death due to MS), with a focus on functional system scores (pyramidal, cerebellar, brainstem, sensory and visual systems) and ambulatory capacity.

Additional functional assessments of Dresden Protocol for Multidimensional Walking Assessment included the timed 25-foot walk test (T25FWT) and the 2-minute walk test, both widely validated tools for assessing short-distance and sustained walking performance, respectively. The T25FWT measures the time taken to walk 25 feet at maximum effort and is sensitive to changes in walking speed over time in people with MS.^29^ The 2-minute walk test evaluates walking endurance by measuring the distance covered in 2 min under standardized conditions.^30^

Patient-reported outcomes were collected, including the Multiple Sclerosis Health Resource Utilisation Survey, a comprehensive tool for evaluating resource utilization by pwMS^31^; the 12-Item Multiple Sclerosis Walking Scale, a self-reported measure assessing the impact of MS on walking ability^32^; and the Early Mobility Impairment Questionnaire, a validated tool for screening mobility problems in MS, often preceding clinical disability.^33^

### Clustering and analytical approach

#### Feature selection

The collected numerical data exhibited varying ranges across different features and participants, necessitating appropriate scaling. These were normalized through consequent item filtering, data ingestion and Z-scaling. To ensure the model's clinical relevance and accuracy, we integrated both established medical knowledge and data-driven techniques in our feature selection process. This combined approach prioritized features known to be clinically significant indicators of gait clusters, while also leveraging statistical methods to identify the most informative variables from our dataset.

Feature selection was carefully guided by existing literature and clinical relevance beyond relying solely on automated approaches.^12,34^ This balanced approach was employed to ensure the inclusion of meaningful, interpretable variables directly linked to gait and MS pathology, while preserving sufficient data complexity to allow for the identification of novel patterns and potential subgroup distinctions. Thus, feature selection was optimized to minimize redundancy and noise while maintaining exploratory potential in unsupervised learning, reducing prediction bias, streamlining the training process and enhancing the overall robustness and interpretability of the analysis.

Following feature selection, we performed feature extraction to further refine the dataset. To evaluate the relationships among features, a feature Pearson correlation matrix was computed (Supplementary Fig. 1), providing insight into the relationships between variables. Features showing high collinearity (absolute values > 0.8) were assessed and selectively retained or removed to reduce noise and optimize model performance, as high correlations are commonly considered indicative of strong collinearity that can negatively impact multivariate analyses. Given the multidimensional nature of the data, which included numerous variables from both gait assessments and MRI, we employed principal component analysis (PCA) as a dimensionality reduction technique. PCA is a powerful statistical method that transforms a large set of potentially correlated variables into a smaller set of uncorrelated variables called principal components (PCs). These PCs capture the majority of the variance, or information, present in the original dataset. This step was necessary to counteract the curse of dimensionality, which can render statistical methods, such as clustering, ineffective in high-dimensional data spaces by increasing sparsity and reducing meaningful differentiation between observations. Thus, this enabled the retention of all selected features while leveraging their interdependencies to maximize explanatory power, while ensuring that potential interactions among variables were preserved.

#### ML algorithms

Several established clustering algorithms, such as k-means, DBSCAN and hierarchical clustering, were applied to gait and MRI features after dimensionality reduction through PCA.^19,35^ To determine the optimal clustering algorithm and number of clusters, we evaluated Silhouette scores, Calinski–Harabasz scores and Davies–Bouldin scores for different combinations of PCA components and clustering approaches (Supplementary Fig. 2). The Silhouette Score measures cluster cohesion and separation, with higher values indicating a better clustering quality while in case of the Davies–Bouldin score the opposite holds true i.e. lower values indicate a better quality. The Calinski–Harabasz Score evaluates cluster density where a higher density is considered better. We further tested k-means clustering with varying numbers of clusters (3, 4 and 5 clusters) to identify the optimal grouping. The 4-cluster solution demonstrated a balance between distinct cluster separation and clinical interpretability.

The final model selected used k-means clustering with four clusters and the first three PCs.^19,35,36^ K-means is one of the most widely used clustering algorithms in unsupervised ML.^36^ It partitions data into a predefined number of clusters, ‘k’, by minimizing the variance within each cluster. The algorithm operates iteratively, initially assigning random centroids, followed by assigning each data point to the nearest centroid and recalculating the centroid positions based on the updated cluster memberships. This process repeats until the centroids stabilize or a convergence criterion is met.

#### Statistical evaluation of cluster differences

To explore inter-cluster differences in clinical measures (e.g. EDSS scores, disease duration or functional system scores), gait parameters, and MRI-derived features, descriptive statistics were computed. Continuous and ordinal variables were summarized using mean ± SD or medians and interquartile ranges (IQR), as appropriate. Frequencies and percentages were reported for categorical variables. Differences across the four clusters were tested using the nonparametric Kruskal–Wallis test. Effect sizes were calculated using the nonparametric eta-squared (*η*²) to assess the magnitude of differences between clusters.^37^  *Post hoc* pairwise comparisons were conducted using Dunn’s test with Bonferroni correction to adjust for multiple comparisons. Chi-square tests were used to assess differences in proportions across clusters. Effect sizes were calculated here using Cramér's V.^37^  *Post hoc* pairwise comparisons of proportions were conducted using the Bonferroni correction for multiple testing.

**Figure 1:**
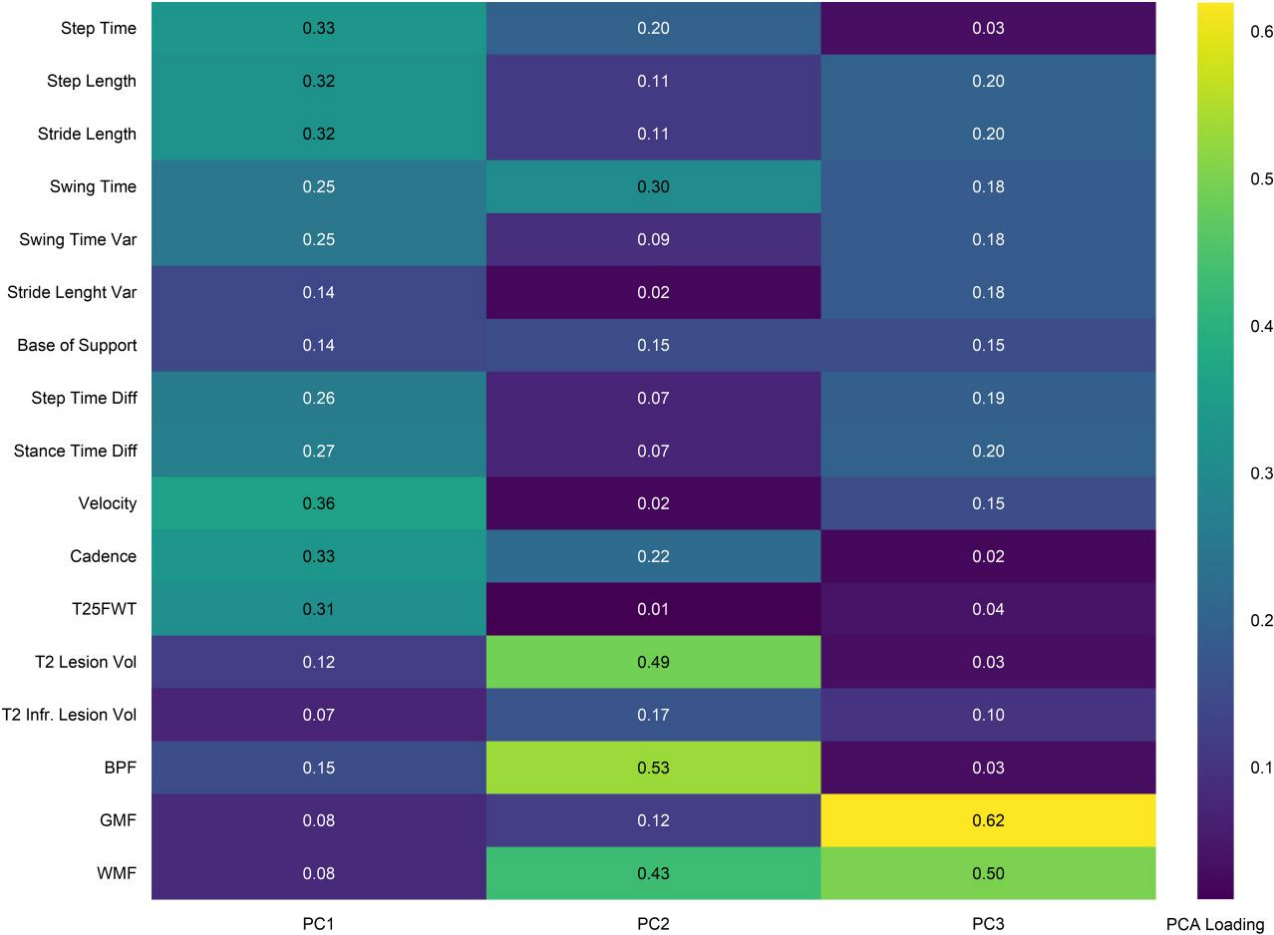
**PCA loadings of gait and MRI metrics.** The heatmap illustrates the absolute PCA loading values of each standardized gait and MRI variables on the first three PCs derived from the dataset (*N* = 1026). Higher absolute values indicate stronger contribution to each component. Variability (Var) measures derive from SD assessment. Gait parameters (e.g. velocity, cadence) exhibit higher loadings in PC1. MRI features, including BPF and T2 lesion volume, contribute mainly to PC2. PC3 predominantly captures structural brain changes, with higher contributions from grey parenchymal fraction and white matter fractions. Diff: difference; T25FWT, timed 25-foot walk test; Vol, volume; Infr, infratentorial.

Clustering analyses were performed using the scikit-learn ML library in Python. All computations were conducted on an Acer Nitro AN515-58 laptop with a 12th Gen Intel Core i7-12700H processor (2.7 GHz, 14 cores) and 16 GB RAM. Statistical significance was set at *P* < 0.05 and all statistical tests were two-tailed. Statistical analysis was conducted using SPSS (V30, IBM Corp., Armonk, NY, USA). Figure 1 was produced with GraphPad Prism 7 (GraphPad Software, Inc., CA, USA).

## Results

A total of 1026 pwMS were included in the analysis. Detailed clinical, and treatment characteristics are summarized in Table 1. The mean age of the cohort was 45.2 ± 13.6 years, with a majority being female (72.6%). Most pwMS had relapsing-remitting multiple sclerosis , with a median EDSS of 2.5 (IQR: 1.5–3.5), and disease duration of 8 years (IQR: 6–12).

**Table 1:** Demographic, clinical and mobility characteristics (*N* = 1026)

Age (years, mean, SD)	45.2 ± 13.6
Sex	
Female (*n*, %)	676 (72.6%)
Height (cm, mean, SD)	169.8 ± 13.9
Weight (kg, mean, SD)	74.5 ± 16.7
BMI (cm^2^/kg, mean, SD)	24.5 (5.5)
Disease course	
Relapsing MS (RMS)	919 (89.6%)
CIS (*n*, %)	14 (1.4%)
RRMS (*n*, %)	905 (88.2%)
Progressive MS (PMS)	107 (10.5%)
PPMS (*n*, %)	52 (5.1%)
SPMS (*n*, %)	55 (5.4%)
Disease duration (years, median, IQR)	8 (6–12)
EDSS (median, IQR)	2.5 (1.5–3.5)
Brainstem FSS (median, IQR)	1.0 (0.0–1.0)
Pyramidal FSS (median, IQR)	1.0 (1.0–2.0)
Cerebellar FSS (median, IQR)	1.0 (0.0–2.0)
T25FWT (s, mean, SD)	5.29 ± 2.25
2MWT (m, mean, SD)	151.12 ± 31.8
DMT (*n*, %)	
No treatment	233 (22.7%)
Low efficacy	290 (28.3%)
High efficacy	489 (47.7%)
Others	14 (1.4%)
EMIQ score (median, IQR)	14.8 (3.7–37.0)
MSWS12 score (median, IQR)	13 (0–38)

Disease modifying treatment (DMT) grouping was performed based on mechanism of action, where high-efficacy therapies included B-cell depletion, natalizumab, S1PR modulators, alemtuzumab and cladribine; low-efficacy therapies included injectable (interferone, glatiramer acetate) and oral (teriflunomide, dimethyl fumarate and diroximel fumarate) baseline therapies. Other therapies included, e.g. inclusion in clinical trials or azathioprine. Disease duration was calculated as the difference between the test date and year of diagnosis. The early mobility impairment questionnaire (EMIQ) and the 12-item multiple sclerosis walking scale (MSWS-12) were used as patient-reported outcomes to assess mobility impairments, where higher scores on both measures indicate greater perceived impairment. Percentages reflect column-valid proportions. BMI, body mass index; CIS, clinically isolated syndrome; PPMS, primary progressive multiple sclerosis; SPMS, secondary progressive multiple sclerosis; T25FWT, timed 25-foot walk test; 2MWT, 2-minute walk test.

### Feature analysis

As part of our preliminary data exploration, we assessed the correlation between all included gait and MRI-derived features to understand the relationships within the dataset and guide subsequent dimensionality reduction and clustering steps. The correlation matrix is shown in Supplementary Fig. 1. Strong correlations were observed between spatiotemporal gait metrics, such as step length and stride length (*r* = 1.00), or cadence and velocity (*r* = 0.91). Similarly, step time showed a strong negative correlation with cadence (*r* = −0,92) and velocity (*r* = −0.75). Considering MRI-derived features, BPF was moderately negatively correlated with T2 lesion volume (*r* = −0.65). Low to negligible correlations were noted between gait and MRI features.

The three first PCs were selected, which explained a significant proportion of the variance in the dataset. PC1 primarily captured gait-related parameters, with high loadings for velocity, cadence, and spatiotemporal parameters such as step time average, stride length average, and swing time average (Fig. 1). While PC2 highlights features linked to lesion burden and brain structure, such as T2 lesion volume, infratentorial T2 lesion volume, and BPF, PC3 predominantly featured grey matter fractions and white matter fractions.

### Brain imaging helped differentiate gait clusters

**Figure 2:**
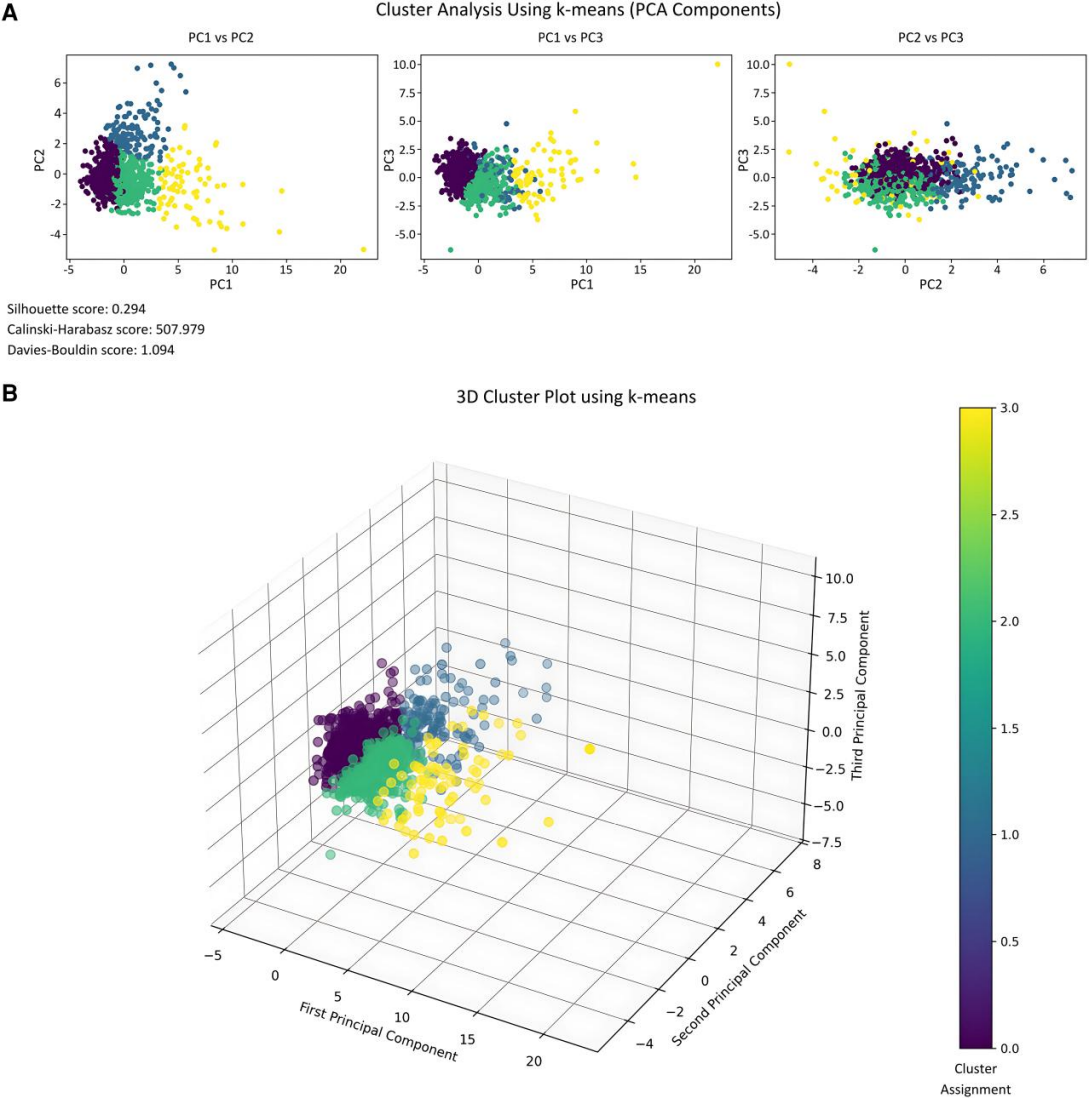
**K-means clusters based on PCA components.** Results of K-means clustering applied to the first three PCs derived from gait and MRI-based features (*N* = 1026). The top panel (**A**) shows 2D scatter plots of the clusters across different PCs combinations (PC1 vs. PC2, PC1 vs. PC3 and PC2 vs. PC3, respectively). The bottom panel (**B**) provides a 3D visualization of the clusters, with colour-coding representing different cluster assignments. The silhouette score (0.294), Calinski–Harabasz score (507.979) and Davies–Bouldin score (1.094) indicate the clustering validity and separability. Purple: Cluster 1 (faster stable gait, *n* = 490). Green: Cluster 2 (slower stable gait, *n* = 331). Blue: Cluster 3 (moderately unstable gait, *n* = 129). Yellow: Cluster 4 (slow severely unstable gait, *n* = 76).

Clustering analysis using k-means on the PCs revealed four distinct clusters (Fig. 2, Supplementary Table 1). The silhouette score of 0.294, the Calinski–Harabasz score of 507.979, and the Davies–Bouldin score of 1.094 suggest moderate separability and compactness of the clusters. Clusters showed clear separation across PC1 and PC2, and partial separation across PC3, reflecting their different profiles.

### Clusters reflected distinct gait profiles

The clusters were further characterized based on spatiotemporal gait parameters, revealing clear distinctions in gait performance across groups (Table 2, Supplementary Table 2).

**Table 2:** Gait features across clusters

Gait feature	1, Faster stable gait (*n* = 490)	2, Slower stable gait (*n* = 331)	3, Moderately unstable gait (*n* = 129)	4, Severely unstable gait (*n* = 76)	*P*-value	Effect size
Velocity (cm/s)	134.2 (13.7)^b,c,d^	104.9 (13.5)^a,d^	106.1 (21.3)^a,d^	55.7 (17.9)^a,b,c^	<0.001	0.532
Cadence (steps/min)	118.6 (7.2)^b,c,d^	105.8 (7.9)^a,c,d^	113.2 (9.7)^a,b,d^	77.8 (16.0)^a,b,c^	<0.001	0.360
Base of support (cm)	9.47 (2.51)^b,c,d^	10.97 (3.04)^a,d^	12.47 (3.81)^a^	13.23 (4.40)^a,b^	<0.001	0.089
Step length (cm)	67.95 (5.58)^b,c,d^	59.49 (6.43)^a,c,d^	56.07 (9.57)^a,b,d^	42.69 (10.03)^a,b,c^	<0.001	0.376
Stride length (cm)	136.38 (11.25)^b,c,d^	119.42 (12.90)^a,d^	112.72 (19.19)^a,d^	85.84 (20.31)^a,b,c^	<0.001	0.376
Step time (s)	0.51 (0.03)^b,c,d^	0.57 (0.04)^a,c,d^	0.53 (0.05)^b,c,d^	0.82 (0.23)^a,b,c^	<0.001	0.358
Swing time (s)	0.39 (0.02)^b,d^	0.42 (0.03)^a,c,d^	0.39 (0.03)^b,d^	0.49 (0.09)^a,b,c^	<0.001	0.358
Step time diff (s)	0.01 (0.01)^b,c,d^	0.02 (0.03)^a,d^	0.03 (0.03)^a,d^	0.14 (0.19)^a,b,c^	<0.001	0.358
Stance time diff (s)	0.01 (0.01)^b,c,d^	0.02 (0.02)^a,d^	0.02 (0.02)^a,d^	0.09 (0.12)^a,b,c^	<0.001	0.115
Swing time var (s)	0.012 (0.006)^b,c,d^	0.021 (0.016)^a,d^	0.020 (0.012)^a,d^	0.082 (0.082)^a,b,c^	<0.001	0.318
Stride length var (cm)	3.92 (1.69)^b,c,d^	4.67 (2.87)^a,c,d^	5.50 (3.22)^a,b,d^	7.40 (4.99)^a,b,c^	<0.001	0.074

Values are presented as mean (SD). Variability measures derive from SD assessment. Statistical significance was assessed using the Kruskal–Wallis test, followed by pairwise comparisons with Bonferroni correction for multiple testing. Effect sizes were calculated using eta-squared (*η*²). Superscripts indicate significant differences between clusters: ^a^*P* < 0.05 compared to Cluster 1 (faster stable gait), ^b^*P* < 0.05 compared to Cluster 2 (slower stable gait), ^c^*P* < 0.05 compared to Cluster 3 moderately unstable gait); and ^d^*P* < 0.05 compared to Cluster 4 (severely unstable gait). Var, variability; Diff, difference.

Cluster 1 (faster stable gait, *n* = 490, 47.8%): Representing the best-performing group, individuals in this cluster exhibited the most efficient and stable gait profiles. PwMS demonstrated the highest velocities, longest stride lengths and lowest stride length variability. Step time and swing time were the shortest among all clusters, reflecting optimal coordination and control. Variability and asymmetry measures were consistently low, and the base of support was narrow, further underscoring their superior stability. *Post hoc* tests confirmed that this cluster was significantly better across all gait parameters compared to the other groups (*P* < 0.001).

Cluster 4 (slow severely unstable gait, *n* = 76, 7.4%): Representing the most impaired group, pwMS in this cluster displayed the slowest velocities, shortest stride lengths and the highest variability and asymmetry measures. Stride length variability and swing time variability were markedly elevated compared to all other clusters, indicating severe instability and poor gait control. Step and stance time differences were also significantly higher, underscoring profound coordination deficits. The base of support was the widest, further reflecting compensatory adjustments for instability. Most parameters were significantly worse compared to the other clusters (*P* < 0.001).

Clusters 2 (slower stable gait) and 3 (moderately unstable gait) demonstrated relatively similar gait features overall, though with notable differences in specific parameters:

Cluster 2 (slower stable gait, *n* = 331, 32.3%): Individuals in this group exhibited moderate gait impairments with a relatively stable profile. Velocity and stride length were lower compared to Cluster 1 (faster stable gait), but step time and variability measures, such as stride length variability, remained significantly better than those observed in Cluster 3 (moderately unstable gait) or 4 (slow severely unstable gait). The base of support was wider than Cluster 1, but narrower than Clusters 3 and 4. Statistical comparisons revealed marked differences in comparison with Cluster 3: people in Cluster 2 took significant longer and fewer steps per minutes, and exhibited lower stride length variability, and had a non-significant tendency towards a narrower base of support.

Cluster 3 (moderately unstable gait, *n* = 129, 12.6%): While people in this group reached similar velocity as people in Cluster 2 (slower stable gait), the gait profile of individuals in Cluster 3 was less efficient and more unstable. Cadence was significantly higher, suggesting that pwMS in this group required more steps per minute to achieve similar velocities, with shorter step lengths. The base of support was wider than those of people in Cluster 1 (faster stable gait) and more closely resembled Cluster 4 (slow severely unstable gait), indicating a shift toward greater instability. Stride length variability was significantly higher than for Cluster 2, reflecting reduced gait efficiency and control.

Overall, the analysis highlights a continuum of clinical gait dysfunction, with Cluster 1 (faster stable gait) demonstrating the best gait performance and stability, Cluster 4 (slow severely unstable gait) reflecting severe instability, and Clusters 2 (slower stable gait) and 3 (moderately unstable gait) representing intermediate profiles. Effect sizes varied across gait features, providing additional insight into the magnitude of inter-cluster differences beyond statistical significance. The largest effect sizes were observed for velocity (*η*² = 0.532), step length (*η*² = 0.376) and cadence (*η*² = 0.360), indicating more marked differences between clusters in these mobility parameters. In contrast, stride length variability (*η*² = 0.074) and base of support (*η*² = 0.089) showed the smallest effect sizes, suggesting more subtle group differences.

### Distinct MRI cluster features

Distinct MRI characteristics emerged when comparing lesion volumes and brain atrophy among gait clusters (Table 3, Supplementary Table 3). Lesion volumes were comparable between Clusters 1 (faster stable gait) and 2 (slower stable gait). In contrast, Clusters 3 (moderately unstable gait) and 4 (slow severely unstable gait) exhibited significantly higher total T2, juxtacortical and periventricular lesion volumes. Notably, Cluster 3 had the highest T2 and infratentorial lesion burdens. When lesion volumes were normalized to brain volume, a similar pattern was observed: Clusters 1 and 2 showed similar values, while Cluster 4 was significantly higher than both, and Cluster 3 demonstrated the highest lesion fraction.

**Table 3:** MRI features across clusters

MRI feature	1, Stable gait (*n* = 490)	2, Slower stable gait (*n* = 331)	3, Moderately impaired gait (*n* = 129)	4, Severely impaired gait (*n* = 76)	*P*-value	Effect size
Total T2 lesion volume (mL)	5.90 (5.44)^c,d^	6.41 (5.63)^c,d^	24.31 (12.56)^a,b,d^	11.84 (10.21)^a,b,c^	<0.001	0.036
Infratentorial T2 lesion volume (mL)	0.04 (0.10)^c,d^	0.05 (0.09)^c^	0.20 (0.43)^a,b^	0.10 (0.16)^a^	<0.001	0.172
Brain parenchymal fraction	0.863 (0.016)^c,d^	0.860 (0.016)^c,d^	0.817 (0.022)^a,b,d^	0.844 (0.023)^a,b,c^	<0.001	0.100
Gray matter fraction	0.484 (0.016)^b,c,d^	0.470 (0.019)^a^	0.465 (0.021)^a^	0.471 (0.018)^a^	<0.001	0.129
White matter fraction	0.379 (0.018)^b,c^	0.390 (0.018)^a,c,d^	0.351 (0.023)^a,b,d^	0.373 (0.024)^b,c^	<0.001	0.036
Total T2 lesion volume fraction	0.005 (0.004)^c,d^	0.005 (0.005)^c,d^	0.022 (0.012)^a,b,d^	0.013 (0.009)^a,b,c^	<0.001	0.151
Thalamic volume (mL)	13.44 (1.42)^c,d^	13.76 (1.55)^c,d^	12.20 (1.44)^a,b^	12.43 (1.30)^a,b^	<0.001	0.054
Cortical grey matter volume (mL)	520.90 (52.67)^b,c,d^	512.03 (52.25)^a,c,d^	473.34 (52.04)^a,b^	477.17 (49.67)^a,b^	<0.001	0.113
Deep grey matter volume (mL)	39.22 (3.67)^c,d^	39.61 (3.87)^c,d^	35.80 (3.96)^a,b^	36.49 (3.79)^a,b^	<0.001	0.049
Thalamic volume fraction	0.010 (0.001)^c,d^	0.010 (0.001)^c,d^	0.009 (0.001)^a,b^	0.009 (0.001)^a,b^	<0.001	0.059
Cortical grey matter fraction	0.373 (0.024)^b,c,d^	0.361 (0.021)^a,c,d^	0.342 (0.021)^a,b^	0.351 (0.023)^a,b^	<0.001	0.113
Deep grey matter fraction	0.028 (0.002)^c,d^	0.028 (0.002)^c,d^	0.026 (0.002)^a,b^	0.027 (0.002)^a,b^	<0.001	0.076
Juxtacortical T2 lesion volume (mL)	0.93 (2.11)^c,d^	0.75 (1.31)^c,d^	2.53 (5.30)^a,b^	1.17 (1.54)^a,b^	<0.001	0.057
Periventricular T2 lesion volume (mL)	4.78 (4.48)^c,d^	5.49 (5.23)^c,d^	20.41 (12.40)^a,b,c^	12.65 (9.84)^a,b,c^	<0.001	0.123
Other T2 lesion volume (mL)	0.19 (0.45)^c,d^	0.26 (1.17)^c,d^	0.68 (3.67)^a,b^	0.19 (0.19)^a,b^	<0.001	0.022

Values are presented as mean (SD). All volume fractions are normalized to total intracranial volume. Statistical significance was assessed using the Kruskal–Wallis test. Significant differences between gait clusters were evaluated using *post hoc* pairwise comparisons with Bonferroni correction. Effect sizes were calculated using eta-squared (*η*²). Superscripts indicate significant differences between clusters: ^a^*P* < 0.05 compared to Cluster 1 (faster stable gait), ^b^*P* < 0.05 compared to Cluster 2 (slower stable gait), ^c^*P* < 0.05 compared to Cluster 3 moderately unstable gait); and ^d^*P* < 0.05 compared to Cluster 4 (severely unstable gait).

Regarding parenchymal fractions, we observed that Cluster 1 (faster stable gait) had significantly higher BPF than Clusters 3 (moderately unstable gait) and 4 (slow severely unstable gait), and its grey parenchymal fraction and cortical grey parenchymal fraction significantly higher than the other groups.

Thalamic volume and thalamic volume fraction were similar between Clusters 1 (faster stable gait) and 2 (slower stable gait), as well as between Clusters 3 (moderately unstable gait) and 4 (slow severely unstable gait).

Effect sizes also varied across MRI features, although they were generally lower than for gait-derived features. The largest effect sizes were observed for infratentorial T2 lesion volume (*η*² = 0.172), total T2 lesion volume fraction (*η*² = 0.151) and grey parenchymal fraction (*η*² = 0.129), suggesting that these variables showed relatively greater variation across clusters. In contrast, ‘other’ T2 lesion volume (*η*² = 0.022), white matter fractions (*η*² = 0.036) and total T2 lesion volume (*η*² = 0.036) showed the smallest effect sizes.

### Sociodemographic and clinical features of clusters

The clusters were analysed based on sociodemographic and clinical characteristics (Table 4). Cluster 4 (slow severely unstable gait) was characterized by older individuals compared to the other clusters. Cluster 3 (moderately unstable gait) had the longest disease durations compared to the other groups. While almost all pwMS in Cluster 1 (faster stable gait) had relapsing MS (98%), this proportion decreased across clusters, with Cluster 4 (slow severely unstable gait) showing a significantly higher proportion of progressive MS (48.7%). The proportion of pwMS on low-efficacy DMTs was lower in Cluster 4 than in the other groups.

**Table 4:** Demographic, clinical and functional characteristics across clusters

Demographic characteristics	1, Faster stable gait (*n* = 490)	2, Slower stable gait (*n* = 331)	3, Moderately unstable gait (*n* = 129)	4, Severely unstable gait (*n* = 76)	*P*-value	Effect size
Age	39 (33–48)^c,d^	46 (37–56)^c,d^	55 (45–63)^a,b^	57 (47–65)^a,b^	<0.001	0.099
Disease duration (years)	5 (1–10)^c,d^	5 (1–11)^c,d^	13 (6–18)^a,b,d^	11 (5–17)^a,b,c^	<0.001	0.055
BMI	24.06 (21.35–26.98)^b^	24.97 (22.64–29.06)^a^	24.62 (22.5–29.04)	24.55 (21.59–29.01)	0.002	0.011
Disease Course						
Relapsing MS	480 (98.0%)^b,c,d^	290 (87.6%)^d^	110 (85.3%)^d^	39 (51.3%)	<0.001	0.237
Progressive MS	10 (2.0%)	41 (12.4%)^a^	19 (14.7%)^a^	37 (48.7%)^a,b,c^		
Sex						
Female	356 (77.9%)^b^	195 (65.7%)	85 (73.3%)	40 (65.6%)	0.002	0.128
Male	101 (22.1%)	102 (34.3%)^a^	31 (26.7%)	21 (34.4%)		
EDSS	2 (1.5–2.5)^b,c,d^	3 (2–4)^a,c,d^	3.5 (2–4.5)^a,b,d^	6 (6–6.5)^a,b,c^	<0.001	0.237
Brainstem FS	0 (0–1)^b,c,d^	1 (0–1)^a,d^	1 (1–1)^a,d^	1 (1–2)^a,b,c^	<0.001	0.067
Pyramidal FS	1 (1–1)^b,c,d^	2 (1–3)^a,d^	2 (1–3)^a,d^	3 (3–3)^a,b,c^	<0.001	0.206
Cerebellar FS	1 (0–1)^b,c,d^	1 (0–2)^a,d^	1 (1–2)^a,d^	3 (2–3)^a,b,c^	<0.001	0.221
DMT						
No Treatment	98 (20.3%)	86 (26.1%)	26 (20.3%)	23 (31.5%)	<0.001	0.101
Low efficacy	164 (34.0%)^d^	88 (26.7%)^d^	32 (25.0%)^d^	6 (8.2%)		
High efficacy	220 (45.6%)	155 (47.1%)	70 (54.7%)	44 (60.3%)		
25-foot walk test	4.29 (3.89–4.74)^b,c,d^	5.03 (4.39–5.94)^a,d^	5.42 (4.63–7.2)^a,d^	11.4 (7.97–17.25)^a,b,c^	<0.001	0.181
2-minute walk test	165.06 (154.06–178.07)^b,c,d^	150.15 (132.34–163.96)^a,d^	143.42 (119.5–160.68)^a,d^	81.7 (60.1–107.5)^a,b,c^	<0.001	0.153
EMIQ score	7 (0–19)^b,c,d^	22 (7–41)^a,c,d^	33 (17–52)^a,b,d^	62 (46–70)^a,b,c^	<0.001	0.197
MSWS-12 score	2 (0–15)^b,c,d^	21 (0–46)^a,c,d^	33 (13–54)^a,b,d^	67 (46–77)^a,b,c^	<0.001	0.177
Help needed in ADL (last 6 months)						
No	438 (89.8%)^b,c,d^	241 (74.4%)^d^	80 (66.1%)^d^	24 (36.4%)	<0.001	0.346
Yes	50 (10.2%)	83 (25.6%)^a^	41 (33.9%)^a^	42 (63.6%)^a,b,c^		
Disability degree						
None	249 (51.7%)^b,c,d^	118 (36.9%)^d^	28 (23.5%)	6 (9.0%)	<0.001	0.254
Recognized disability	146 (30.3%)	111 (34.7%)^d^	33 (27.7%)	12 (17.9%)		
Severe disability	87 (18.0%)	91 (28.4%)^a^	58 (48.7%)^a,b^	49 (73.1%)^a,b,c^		
Changes in properties due to MS						
No	480 (98.4%)^b,c,d^	302 (93.2%)^d^	110 (91.7%)^d^	43 (66.2%)	<0.001	0.323
Yes	8 (1.6%)	22 (6.8%)^a^	10 (8.3%)^a^	22 (33.8%)^a,b,c^		
Family with working hours reduction						
No	481 (99.2%)^c,d^	308 (96.9%)	111 (94.9%)	61 (92.4%)	0.001	0.128
Yes	4 (0.8%)	10 (3.1%)	6 (5.1%)^a^	5 (7.6%)^a^		

Values are presented as median (interquartile range) or absolute values (percentage), as appropriate. Statistical significance was assessed using chi-square tests, followed by pairwise comparisons with Bonferroni correction for multiple testing. Effect sizes were calculated here using Cramer’s V. Disease modifying treatment (DMT) grouping was performed based on mechanism of action, where high-efficacy therapies included B-cell depletion, natalizumab, S1PR modulators, alemtuzumab and cladribine; low-efficacy therapies included injectable (interferone and glatiramer acetate) and oral (teriflunomide, dimethyl fumarate and diroximel fumarate) baseline therapies. The early mobility impairment questionnaire (EMIQ) and the 12-item multiple sclerosis walking scale (MSWS-12) were used as patient-reported outcomes to assess mobility impairments, where higher scores on both measures indicate greater perceived impairment. Percentages reflect column-valid proportions. Self-reported data were obtained regarding help needed in activities of daily living (ADL), disability degree, changes in properties and working hours reduction. Superscripts indicate significant differences between clusters: ^a^*P* < 0.05 compared to Cluster 1 (faster stable gait), ^b^*P* < 0.05 compared to Cluster 2 (slower stable gait), ^c^*P* < 0.05 compared to Cluster 3 moderately unstable gait); and ^d^*P* < 0.05 compared to Cluster 4 (severely unstable gait). BMI, body mass index; EDSS, expanded disability status scale.

EDSS scores increased progressively with gait disability across the clusters, and a similar trend was observed in the functional system scores. Clusters 2 (slower stable gait) and 3 (moderately unstable gait) showed comparable performance in the 25-foot walk test and 2-minute walk test, whereas all other inter-cluster comparisons revealed significant differences. Significant differences were observed in the self-assessments, the Early Mobility Impairment Questionnaire and the 12-Item Multiple Sclerosis Walking Scale, with more impaired pwMS reporting greater impairment of mobility in daily life.

The proportion of individuals needing help with activities of daily living in the previous six months also increased markedly across clusters, as did the proportion of those with severe disability degrees. Changes in properties (e.g. modifications to living spaces or vehicles, use of specialized equipment due to MS) were significantly higher in Cluster 4 (slow severely unstable gait), with a similar trend noted for families experiencing reductions in working hours.

## Discussion

This study offers a novel perspective on mobility impairment in MS by integrating advanced quantitative gait analysis and MRI metrics through ML-driven clustering. We identified four distinct clinical gait clusters, underscoring the heterogeneous nature of gait impairments in MS. Notably, intermediate Clusters 2 (slower stable gait) and 3 (moderately unstable gait) revealed unique combinations of spatiotemporal and MRI-derived features, suggesting varying compensatory mechanisms and structural burdens.

The four-cluster k-means solution achieved an optimal balance between distinct model performance and clinical interpretability. The four identified gait-MRI clusters exhibited distinct mobility profiles with a slight overlap as reflected in higher Silhouette and Calinski–Harabasz scores, suggesting a continuum of gait impairment severity in pwMS. Cluster 1 (faster stable gait) exhibited efficient gait and preserved brain structures, while Cluster 4 (slow severely unstable gait) showed severe instability and poor motor control.^11,38^ Although velocity remained relatively similar between Clusters 2 and 3, Cluster 3 exhibited a compensatory gait with higher cadence, reduced step length and wider base of support. While experienced clinicians may intuitively identify patients in transition, our ML-based approach may add objectivity and detect subtle patterns across multimodal data that may escape conventional assessments. Moreover, exploratory analyses including the 2-minute walk test revealed additional discriminatory value in walking endurance across clusters, supporting its inclusion in future integrative models. The distinct effect sizes of the gait parameters underscore the discriminatory power of specific metrics in distinguishing gait profiles, with higher effect sizes for velocity and step length. Interestingly, base of support and stride length variability showed lower effect sizes. These findings highlight the robustness of certain gait metrics in clustering pwMS based on mobility impairment while underscoring the multidimensional nature of gait dysfunction.

Allowing participants to walk at their preferred speed has become widely used due to its relevance to everyday life and the varied demands it places on maintaining postural control at different speeds. However, standardized walking speeds, such as those based on the T25FWT or fixed velocities, may also provide valuable insights and may facilitate comparisons across pwMS. Filli *et al*.^25^ have emphasized the advantages of homogenous speeds for identifying kinematic patterns in MS, while our study explored gait at preferred speeds to better reflect real-world conditions and compensatory strategies.

PCA revealed separable contributions of gait and MRI features. PC1 primarily captured gait efficiency metrics, while PC2, in contrast, encapsulated lesion burden and rhythm-associated variables (temporal measures), suggesting a link to radiological disease burden. PC3 highlighted structural brain changes, such as variations in grey and white matter fractions. Overall, clusters with higher lesion burdens and reduced BPF tended to show more severe gait impairments, aligning with clinical expectations and previous literature.^39-41^ However, the correlations between brain MRI features and gait parameters were relatively low. The moderate separability of clusters may suggest that gait impairments in MS may result from complex, multidimensional interactions, driven by a combination of network-level disruptions and lack of compensatory mechanisms, together with lesion localization and global volumetric measures.^42^ This supports the multifactorial nature of gait impairment in MS and highlights the clinico-radiological paradox that has been described in MS. Lesion localization, especially infratentorial involvement, could contribute disproportionately due to its effects on balance and motor coordination.^4,43-46^

Certain limitations must be acknowledged. First, moderate silhouette scores suggest some overlap between clusters despite satisfactory cluster separation. This may, however, reflect the nature of MS as a continuum and the inherent complexity and heterogeneity of gait dysfunction in this disease. Second, the cross-sectional design limits conclusions about disease trajectories. Longitudinal changes, including clustering of gait change profiles, are warranted to evaluate predictive value. A further limitation is the variable time interval between MRI and gait assessment (median 69 days, IQR 36–123), which may introduce noise in structure-function correlations; however, this is mitigated by our focus on slowly evolving structural markers such as lesion burden and atrophy. In future prospective studies, this interval should be restricted (e.g. ≤30 days to improve temporal alignment). Third, our cohort, while representative of a specialized MS centre, may not capture the full spectrum of gait impairments. As a referral centre, several pwMS underwent the examination already on advanced stages of their disease with a relatively long median disease duration and already established DMT. Additionally, we did not include in the clustering analysis tests from symptom domains such as fatigue, cognitive dysfunction, or psychosocial factors, which are important contributors of gait impairment. Finally, the monocentric design may limit the generalizability of our findings. As the gait technology used is not yet widely available in routine practice, external validation in broader, multi-centre cohorts is essential.

A key strength of this study is the integration of clinical, gait, and imaging real-world data in a standardized evaluated cohort using robust clustering techniques. This multimodal approach enabled the identification of clinically meaningful mobility phenotypes in pwMS beyond what can be easily captured through conventional assessments. While clinical evaluation by caregivers remains central in assessing gait impairment, our approach offers a complementary, objective perspective. Although further research is needed, identifying distinct gait-MRI clusters may offer promising opportunities for early treatment and rehabilitation strategies to individual needs. For example, pwMS from Cluster 3 showed structural MRI abnormalities and altered gait patterns suggestive of emerging instability and may benefit more from intensive physiotherapy training (e.g. at daily sessions or intensive inpatient rehabilitation) to improve balance and coordination, while individuals from Cluster 1 may be suited for a maintenance or fitness-based program.

Further studies should explore multidimensional gait-MRI profiles in selected cohorts (e.g. early MS, low disability degree) to identify and stratify pwMS who may benefit more from early intervention strategies. The workflow presented here can be readily adopted by other sites that collect instrumented walkway or wearable-sensor data, provided that spatiotemporal variables are extracted in a comparable manner and linked to routinely acquired MRI metrics. Moreover, the methodological approach could be adapted for gait phenotyping in other neurological disorders such as Parkinson’s disease or stroke, offering a framework for digital multimodal mobility biomarkers across conditions. Looking ahead, data-driven models incorporating additional modalities may serve as foundation for establishing a framework for ‘gait phenotypes’ and for developing personalized disease simulations, so-called digital twins, to predict progression and tailor interventions.^47^

## Supplementary Material

fcaf381_Supplementary_Data

## Data Availability

The datasets generated and analysed during this study are not publicly available due to institutional and ethical restrictions but are available from the corresponding author upon reasonable request. The custom code used in this analysis is publicly available at https://github.com/stefanieZhao77/Cluster-Mate.
