# Supplementary material for: Machine learning integration of MRI and gait reveals mobility phenotypes in multiple sclerosis
Source: Brain Commun. 2025 Oct 6;7(5):fcaf381. doi: 10.1093/braincomms/fcaf381 (PMC12529069; doi:10.1093/braincomms/fcaf381)
Supplement: fcaf381_Supplementary_Data [file fcaf381_supplementary_data.pdf]

Supplementary Material

Supplementary Figure 1. Feature Correlation Matrix

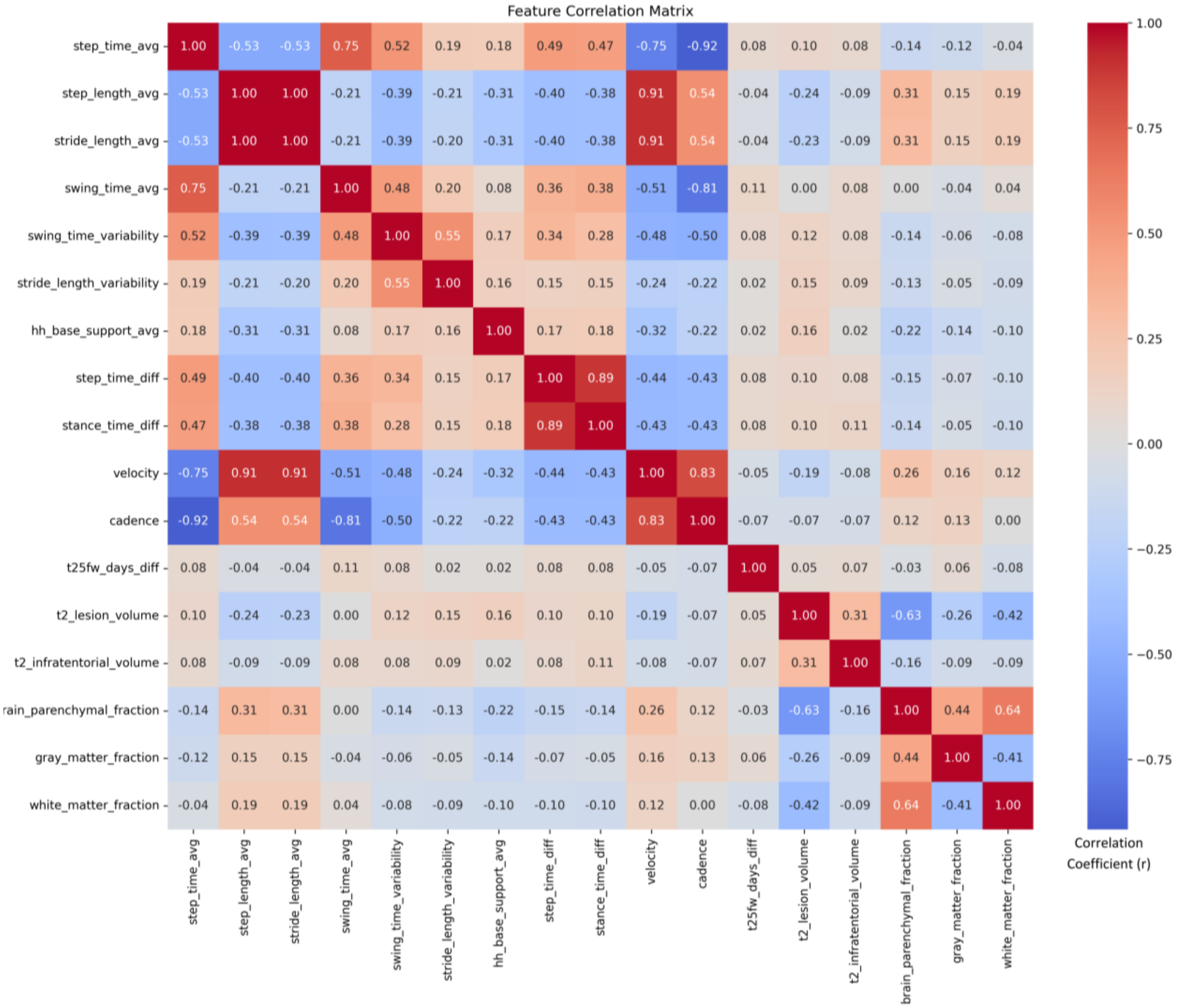

Supplementary Figure 1. The feature correlation matrix illustrates the relationships between the features used in the clustering analysis. Correlation values range from -1 (strong negative correlation) to +1 (strong positive correlation), with a score of 0 denoting no correlation. Pairwise relationships were computed using the Pearson correlation coefficient (r). This analysis was based on 1026 people with multiple sclerosis. The matrix was computed as part of the feature selection process to minimize redundancy and improve the interpretability of the dataset. Features showing high collinearity (absolute values > 0.8) were carefully evaluated to reduce noise and optimize model performance.

Supplementary Figure 2. Clustering Performance across PCA Components and Algorithms

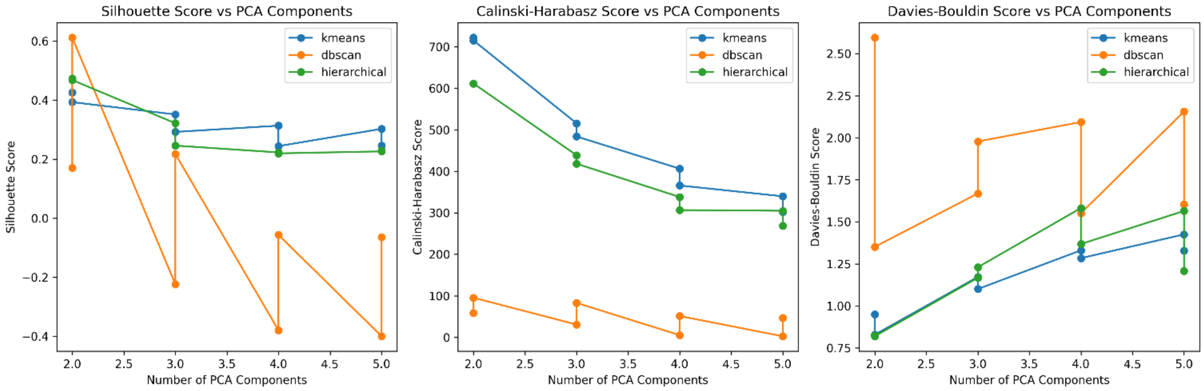

Supplementary Figure 2. Comparison of Silhouette score, Calinski-Harabasz score, and Davies-Bouldin score for k-means, DBSCAN, and hierarchical clustering algorithms across different numbers of PCA components. The 4-cluster solution using k-means with three principal components was chosen based on its balance of clustering performance and interpretability.

Supplementary Table 1. Principal Component Distributions Across Gait-MRI Clusters

Principal Component	1, Faster stable gait (n=490)	2, Slower stable gait (n=331)	3, Moderately unstable gait (n=129)	4, Severely unstable gait (n=76)	*p*-Value
**PC 1**	-1.552 (0.754)[b.c.d]	0.436(0.983)[a.d]	1.088(1.554)[a.d]	6.263(2.980)[b.c.d]	< 0.001
**PC 2**	-0.209(0.891)[b.c]	-0.570(0.907)[a.c]	2.814(1.328)[a.b.d]	-0.947(1.715)[c]	< 0.001
**PC 3**	0.429(0.871)[b.c]	-0.681(1.033)[a.c.d]	-0.055(1.260)[a.b]	0.290(2.057)[b]	< 0.001

Supplementary Table 1. Statistical significance was assessed using the Kruskal-Wallis test, followed by pairwise comparisons with Bonferroni correction for multiple testing. Superscripts indicate significant differences between clusters: [a] $p < 0.05$ compared to Cluster 1 (faster stable gait), [b] $p < 0.05$ compared to Cluster 2 (slower stable gait), [c] $p < 0.05$ compared to Cluster 3 (moderately unstable gait); and [d] $p < 0.05$ compared to Cluster 4 (severely unstable gait).

Supplementary Table 2. Gait Parameters Across Gait-MRI Clusters

Gait Feature	1, Faster stable gait (n=490)	2, Slower stable gait (n=331)	3, Moderately unstable gait (n=129)	4, Severely unstable gait (n=76)
Velocity (cm/s)	133.00 (124.70-142.80)	106.50 (95.90-114.20)	108.80 (89.30-120.30)	57.30 (43.70-67.40)
Cadence (steps/min)	118.30 (113.70-123.00)	105.80 (100.40-110.60)	113.50 (107.60-119.80)	79.60 (68.20-88.40)
Base of Support (cm)	9.26 (7.73-10.97)	10.82 (8.64-13.06)	11.95 (9.95-14.83)	13.54 (10.18-16.31)
Step Length (cm)	67.39 (64.37-71.43)	59.68 (56.03-63.28)	57.49 (49.99-62.18)	42.66 (35.95-49.60)
Stride Length (cm)	135.31 (129.04-143.66)	119.83 (112.33-126.87)	115.26 (100.53-125.09)	86.06 (72.22-99.49)
Step Time (s)	0.51 (0.49-0.53)	0.57 (0.54-0.60)	0.53 (0.50-0.56)	0.75 (0.68-0.88)
Swing Time (s)	0.39 (0.38-0.41)	0.42 (0.40-0.44)	0.39 (0.37-0.41)	0.48 (0.43-0.55)
Step Time Diff (s)	0.01 (0.00-0.01)	0.01 (0.01-0.03)	0.01 (0.01-0.03)	0.11 (0.04-0.15)
Stance Time Diff (s)	0.01 (0.00-0.01)	0.01 (0.01-0.02)	0.01 (0.01-0.03)	0.07 (0.03-0.11)
Swing Time Var (s)	0.01 (0.01-0.01)	0.02 (0.01-0.02)	0.02 (0.01-0.03)	0.05 (0.04-0.10)
Stride Length Var (cm)	3.67 (2.92-4.53)	3.99 (3.20-5.03)	4.64 (3.81-6.36)	5.55 (4.47-8.65)

Supplementary Table 2. Values are presented as median (interquartile range). Variability measures derive from standard deviation assessment. Var: Variabilitiy. Diff: Difference.

Supplementary Table 3. MRI-Derived Parameters Across Gait-MRI Clusters

MRI Feature	1, Stable Gait (n=490)	2, Slower Stable Gait (n=331)	3, Moderately Impaired Gait (n=129)	4, Severely Impaired Gait (n=76)
Total T2 lesion volume (mL)	4.12 (2.25–7.91)	4.64 (2.48–8.38)	22.83 (13.88–31.85)	8.78 (3.38–17.69)
Infratentorial T2 lesion volume (mL)	0.0049 (0.0000–0.0383)	0.0094 (0.0000–0.0527)	0.0415 (0.0062–0.1827)	0.0304 (0.0049–0.1435)
Brain parenchymal fraction	0.864 (0.852–0.874)	0.861 (0.851–0.872)	0.820 (0.804–0.830)	0.846 (0.830–0.861)
Gray matter fraction	0.485 (0.474–0.494)	0.473 (0.458–0.481)	0.466 (0.452–0.479)	0.471 (0.457–0.482)
White matter fraction	0.379 (0.367–0.391)	0.389 (0.378–0.401)	0.353 (0.336–0.369)	0.375 (0.355–0.392)
Total T2 lesion volume fraction	0.0035 (0.0019–0.0065)	0.0040 (0.0021–0.0067)	0.0209 (0.0123–0.0273)	0.0098 (0.0055–0.0174)
Thalamic volume (mL)	13.39 (12.50–14.29)	13.68 (12.68–14.60)	12.18 (11.22–13.17)	12.48 (11.53–13.38)
Cortical gray matter volume (mL)	520.98 (486.12–555.56)	506.22 (477.90–545.05)	469.38 (439.15–508.62)	470.32 (447.88–501.90)
Deep gray matter volume (mL)	38.85 (36.81–41.41)	39.42 (36.87–41.97)	35.73 (33.28–37.98)	37.09 (34.09–38.80)
Thalamic volume fraction	0.0096 (0.0091–0.0101)	0.0097 (0.0092–0.0102)	0.0088 (0.0083–0.0093)	0.0091 (0.0086–0.0096)
Cortical gray matter fraction	0.373 (0.359–0.386)	0.361 (0.348–0.376)	0.340 (0.326–0.358)	0.352 (0.334–0.364)
Deep gray matter fraction	0.0280 (0.0270–0.0291)	0.0280 (0.0270–0.0291)	0.0260 (0.0245–0.0272)	0.0270 (0.0256–0.0278)
Juxtacortical T2 lesion volume (mL)	0.39 (0.16–0.98)	0.40 (0.16–0.88)	1.20 (0.68–2.02)	0.91 (0.37–1.36)
Periventricular T2 lesion volume (mL)	3.32 (1.73–6.55)	3.93 (1.99–7.30)	18.91 (10.77–27.27)	9.81 (5.70–18.20)
Other T2 lesion volume (mL)	0.08 (0.02–0.19)	0.08 (0.02–0.21)	0.13 (0.07–0.26)	0.14 (0.05–0.27)

Supplementary Table 3. Values are presented as median (interquartile range). All volume fractions are normalized to total intracranial volume.
